# Expanded Carrier Screening: Current Evidence and Future Directions in the Era of Population Genomics

**DOI:** 10.3390/genes17010058

**Published:** 2026-01-05

**Authors:** Lucrezia Pilenzi, Vincenzo Scorrano, Sara Di Rado, Carlotta Buccolini, Roberta Giansante, Laura Siciliani, Liborio Stuppia, Valentina Gatta, Antonio Capalbo

**Affiliations:** 1 Department of Neuroscience, Imaging and Clinical Sciences, School of Medicine and Health Sciences, “G. d’Annunzio” University of Chieti-Pescara, 66100 Chieti, Italy; lucrezia.pilenzi@studenti.unich.it (L.P.); vincenzo.scorrano@unich.it (V.S.); sara.dirado@phd.unich.it (S.D.R.); carlotta.buccolini@studenti.unich.it (C.B.); roberta.giansante@phd.unich.it (R.G.); laura.siciliani@phd.unich.it (L.S.); stuppia@unich.it (L.S.); v.gatta@unich.it (V.G.); 2 Unit of Molecular Genetics, Center for Advanced Studies and Technology (CAST), “G. d’Annunzio” University of Chieti-Pescara, 66100 Chieti, Italy; 3 Department of Medical, Oral and Biotechnological Sciences, “G. d’Annunzio” University of Chieti-Pescara, 66100 Chieti, Italy

**Keywords:** expanded carrier screening, reproductive genetics, genetic counseling, variants of uncertain significance, artificial intelligence

## Abstract

Expanded carrier screening (ECS) has emerged as a cornerstone of reproductive genetics, enabling the identification of couples at risk of transmitting autosomal recessive and X-linked disorders. Advances in next-generation sequencing and the increasing adoption of exome- and genome-based strategies have greatly expanded its clinical scope. However, despite existing national and international recommendations, substantial heterogeneity remains in gene panel composition, inclusion criteria, and interpretation frameworks. A key novelty of the current genomic era is the availability of large, publicly accessible human genome datasets encompassing broader ancestral diversity. These resources are transforming our understanding of variant frequencies and disease penetrance across populations, supporting the development of evidence-based and ancestry-informed gene panels. In parallel, recent studies investigating the genetic contribution of pathogenic variants to euploid pregnancy losses are opening new perspectives for ECS. Incorporating genes associated with lethal conditions in utero may enhance the predictive power of screening and deepen our understanding of reproductive outcomes, while also demanding careful consideration of clinical validity and counseling implications. This mini-review synthesizes current evidence on ECS, examines ongoing interpretive and implementation challenges, and discusses how population-scale genomic resources and emerging data on reproductive lethality are shaping the next generation of evidence-based carrier screening.

## 1. Introduction

**Figure 1:**
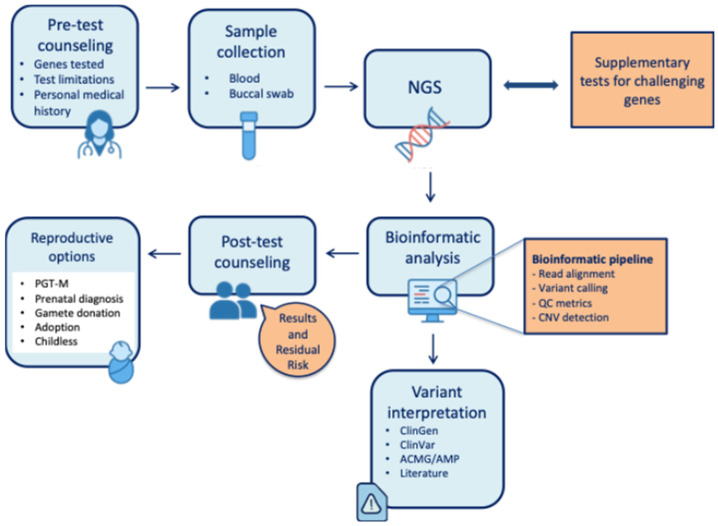
Schematic overview of the carrier screening process integrating pre- and post-test counseling, next-generation sequencing (NGS), variant interpretation, and reproductive decision-making pathways.

Carrier screening (CS) is a genetic test designed to identify pathogenic variants associated with autosomal or X-linked severe childhood onset diseases. CS can be performed on individuals and couples of reproductive ages, even if there is no family history of genetic diseases [1], because most carriers and at-risk couples (ARCs) are asymptomatic and lack a relevant family history. Knowing one’s carrier status can help assess the risk of passing on genetic disorders to one’s children. CS is therefore a fundamental preventive tool, as it allows couples to understand their genetic risks and make informed decisions about their reproductive choices and family planning. For a couple with an increased risk for a specific disease, reproductive choices may vary, including (I) the use of assisted reproductive technology (ART) with preimplantation diagnosis for monogenic diseases (PGT-M), (II) natural conception with subsequent invasive prenatal diagnosis, (III) gamete donation, and (IV) adoption (Figure 1), having a child without screening and accepting the risk of a recessive condition or stay childless [2]. In their initial implementation, carrier screening programs were limited to specific ethnic groups with a relatively high prevalence of certain genetic disorders, such as Tay–Sachs disease among Ashkenazi Jewish populations [3]. In the latter case, the CS is also referred to as “ancestry-based”. In the past few years, rapid advances in next-generation sequencing (NGS) techniques have made it possible to extend testing from single conditions to broader panels, promoting more cost-effective and equitable pan-ethnic expanded carrier screening (ECS) applications. The clinical implementation of expanded carrier screening (ECS) has been marked by considerable debate, largely due to the lack of standardization in gene panel design. This heterogeneity has led to substantial inconsistencies among testing providers, ultimately undermining the clinical value and utility of ECS. In several instances, panels have included genes with uncertain disease associations, low penetrance, or mild phenotypic manifestations, thereby complicating result interpretation and clinical decision-making. The selection of genes for ECS panels requires a careful balance between multiple factors, including the population frequency of the condition, its clinical severity, and the penetrance of the associated variants. Typically, ECS panels encompass genes implicated in inherited metabolic disorders, neurodevelopmental syndromes, cystic fibrosis, muscular dystrophies, and other conditions associated with significant morbidity. While some extended panels now include hundreds of genes, providing a more comprehensive assessment of reproductive risk, this expansion also increases interpretative complexity and emphasizes the importance of robust pre- and post-test genetic counseling. Despite efforts by international and national professional societies to issue recommendations and harmonize ECS practice [4,5,6], consensus on gene selection and implementation strategies remains elusive. This review aims to critically examine the key challenges and evolving frameworks surrounding carrier screening and to encourage reflection on its future integration with advancing genomic technologies, ethical considerations, and psychosocial dimensions. In particular, we discuss how the growing availability of large-scale, publicly accessible genomic repositories [7] (is reshaping the landscape of ECS—offering new opportunities for data-driven standardization across ancestries and potentially enhancing its clinical validity, utility, and scope, including the incorporation of genes associated with severe or lethal phenotypes.

## 2. Gene Selection Criteria in Expanded Carrier Screening

The growing availability of technologies, particularly platforms based on next generation sequencing (NGS), requires careful consideration of the criteria for including genes in diagnostic panels. The selection of target genes is not a simple technical step: it determines the clinical sensitivity and the clinical utility of the test, as well as the ethical and psychological implications for couples undergoing screening. Clinical sensitivity refers to the ability of the screening test to detect carriers of disease-causing variants—essentially, how effectively the chosen genes and variants capture the true carrier status within the population. Regarding the clinical sensitivity of an ECS panel, genetic diversity must be taken into account, as ancestry has a substantial impact on test sensitivity. The distribution of pathogenic variants varies significantly between different populations due to phenomena such as the founder effect, genetic drift, or demographic bottlenecks in ancestral populations. In this regard, leading scientific societies agree on the need for pan-ethnic approaches based on sequencing and more representative databases [8], such as gnomAD v4.1, which is widely used in data-driven carrier-screening panel development and offers broad multi-ancestry representation. A panel with higher clinical sensitivity reduces the chance of false-negative results and more reliably identifies couples at increased reproductive risk. Clinical utility, in contrast, describes how useful the test results are in guiding meaningful medical or reproductive decisions. It encompasses whether the information provided leads to actions that improve outcomes—such as informed reproductive planning, access to early interventions, or avoidance of unnecessary anxiety. Panels with high clinical utility provide results that are not only accurate but also actionable and relevant to the needs and values of patients. Not all genes are equivalent in terms of clinical relevance, reproductive impact, or predictive value. There is considerable variability in the types of ECS offered to couples, particularly in terms of the type and number of genes included in the NGS panel [9]. To improve consistency and clinical relevance, the Italian Society of Human Genetics (SIGU) has established that the selection of genes to be included must follow the following criteria: I the disease must be associated with a well-defined criterion; II the disease must have a negative effect on quality of life/life expectancy; III the disease must cause cognitive and/or physical deficits; IV the disease must require medical and/or surgical intervention; V the pathology must have an early onset; VI an adequate prenatal diagnosis/pre-implantation genetic testing pathway must be available, VII; the frequency of carriers in the reference population must be taken into account [5]. Several studies (Table 1) [10,11,12,13,14,15,16,17,18,19,20,21,22] have shown that panel size directly influences the probability of identifying couples at risk. Feldman et al. (2024) [20] found that almost half of at-risk couple’s cases would have been missed using standard ethnicity-based panels, highlighting the advantage of larger panels. The greater the number of genes, the greater the interpretative complexity: the analysis of rare variants or genes with incomplete expressivity can lead to an overestimation of genetic risk or to reproductive decisions based on partial information. For this reason, some scientific societies, such as the American College of Medical Genetics (ACMG) [5] and the European Society of Human Genetics (ESHG) [6] and American College of Obstetricians and Gynaecologists (ACOG) [23], recommend selection criteria that consider not only the severity and prevalence of the disease, but also clinical validation and the impact on the couple. Specifically, ACOG proposes inclusion criteria for genes similar to SIGU criteria (frequency ≥ 1/100, severe phenotype, necessary interventions, early onset, prenatal diagnosis available) but universally recommends only testing for cystic fibrosis [23]. The ESGH recommends using evidence-based criteria for the inclusion of diseases, such as severity, clinical utility and genes that are adequately characterized to enable reliable classification of variants and standardization of panels to ensure consistency [6]. The ACMG introduces a tiered approach that stratifies conditions based on carrier frequency and suggests adopting tier 3 (cystic fibrosis, spinal muscular atrophy, ”risk-based screening”, conditions with carrier frequency ≥ 1 in 200, carrier frequency and X-linked conditions with prevalence ≥ 1 in 40,000) as the generally applicable standard [5]. Despite differences between scientific societies, they all agree on maintaining a balance between clinical utility, fairness and appropriateness of the genes to be included in the panels. Based on the latest studies [24,25] using the gnomAD v4.1.0 database, which is based on the analysis of the exome of over 700,000 individuals from eight ancestral populations, it is now possible to systematically estimate gene carrier frequencies (GCF). Thanks to the large size of the genomic cohort, the study by Schmitz et al. (2025) [25], identified GCF values for 2987 genes. From the frequency of P/LP variants in these genes associated with autosomal recessive conditions, a list of 286 genes associated with at least moderate severity was derived, meeting the ACMG-recommended carrier frequency criterion of ≥1/200 [5]. This list is only a starting point; the identified genes were subsequently subjected to manual and clinical review, partly due to possible artefacts and therefore distortions in the work. This integrated approach, combining large-scale bioinformatic analysis and expert curation, allows for dynamic updating of carrier screening panels, ensuring that they are truly representative of genetic risk in different populations. The availability of population genomic data with multi-ancestry representation is also offering opportunity to develop equitable and pan-ethnic ECS applications. A useful framework for designing expanded carrier screening (ECS) panels is the analysis of cumulative fetal disease risk (CFDR) curves. Unlike cumulative carrier rate metrics that quantify only the probability that an individual carries a pathogenic or likely pathogenic (P/LP) variant, CFDR provides a more clinically meaningful measure: the probability that a randomly paired couple will conceive an affected fetus due to autosomal recessive or X-linked conditions included in the panel [13]. CFDR therefore integrates both carrier frequency and the inheritance pattern to estimate the actual disease risk at the population level. In a CFDR curve, genes are ordered on the x-axis by their individual contribution to fetal disease risk (typically a function of allele frequency and mode of inheritance), while the y-axis represents the cumulative proportion of total fetal disease risk captured as additional genes are added. The curve typically shows a steep initial rise, reflecting a small number of genes with disproportionately high contributions to CFDR, followed by a gradual plateau where additional, rarer genes add only marginal increments to risk capture. This behavior reflects the well-established long-tail distribution of pathogenic variant frequencies across Mendelian disorders. Recent modelling studies—such as AJHG and GIM recent papers [25,26,27]—demonstrate that selecting genes with a contribution to the fetal disease risk above a defined threshold (e.g., ≥1/1000 CFDR per gene) can efficiently capture the majority of overall risk. Their analysis showed that a panel of 334 genes accounted for approximately 90% of the population’s CFDR, supporting the use of data-driven thresholds to optimize panel design. Moreover, incorporating large-scale population allele frequency datasets such as gnomAD v4.1.0 improves the accuracy of CFDR estimates, ensuring that panels reflect contemporary and ancestrally diverse genomic data. Overall, using CFDR rather than cumulative carrier rate allows for a more clinically relevant and equitable approach to ECS panel construction, ensuring that selected genes meaningfully contribute to fetal disease prevention while avoiding unnecessary expansion into ultra-rare conditions with negligible impact on reproductive risk. Extended carrier screening has clear advantages in identifying at-risk couples who would otherwise not be identified by limited panels, but this requires robust counselling and careful panel design. However, it is not important to underline that the percentage of couples at risk is directly proportional to the number of potentially affected children, which instead indicates the estimated number of births that could actually manifest the genetic condition.

## 3. Interpretation Challenges and Variants of Uncertain Significance (VUS)

The use of extended panels with full gene sequencing based on NGS techniques for the identification of risk couples has increased the sensitivity of the test, but also the possibility of identifying rare variants for which, to date, there is still no clear clinical or functional interpretation. One of the main obstacles to the use of these panels is therefore the interpretative difficulties associated with the identification of variants of uncertain significance (VUS). However, today this aspect is supported by limiting carrier screening panels to well-characterized, highly penetrant, severe, and relatively common recessive childhood conditions, which helps to minimize the incidence and clinical impact of VUS, thereby improving clarity and clinical utility.

In the future, this aspect could be supported by the integration of bioinformatic, functional and segregative data and the use of ML and AI. SIGU and ACMG agree that laboratories performing the analysis should only report genes and variants of clear pathogenicity, specifically only pathogenic (P) (Class 5) or likely pathogenic (LP) (Class 4) variants, while variants of uncertain significance (VUS) should not be reported [8]. VUS may account for approximately 5–10% of findings in expanded carrier screening (ECS), although this proportion can vary widely depending on the ancestral background of the tested population, the specific genes included, and the stringency and granularity of the bioinformatic pipeline used for variant annotation and filtering [28]. Accurate interpretation of VUS requires an integrative approach that combines computational predictions, population allele frequency data, and clinical or genetic evidence, following established frameworks such as ACMG/AMP guidelines. In a subset of cases where available evidence remains insufficient, in vitro functional assays or other experimental models may be necessary to clarify the biological consequences of the variant. Functional studies provide direct insights into how a variant affects gene or protein function, thereby improving the ability to reclassify VUS and determine their potential clinical relevance. The continued advancement of machine learning–based variant interpretation tools and the rapid growth of international genomic and phenotypic databases are expected to substantially reduce the proportion of variants of uncertain significance (VUS), thereby improving the diagnostic accuracy and clinical actionability of carrier screening (CS) [29]. Machine learning platforms increasingly integrate multidimensional data—such as evolutionary conservation scores, protein structural modeling, transcriptomic patterns, and splicing predictions—to generate more reliable pathogenicity estimates. Recent developments including proteomic data mining are showing promises to further boost the prediction towards missense variant classification. popEVE is an example of a deep generative model that combines evolutionary data and human population data to estimate the harmfulness of variants on a proteomic scale [30]. Furthermore, developments in the clinical validity of genome sequencing will also further boost test sensitivity by expanding the resolution towards small CNVs, regulatory and deep intronic pathogenic variants analysis [31]. At the same time, the expansion of global genomic resources, including population-specific allele frequency data and curated variant repositories with detailed clinical annotations, enhances the ability to contextualize rare variants and distinguish benign from pathogenic findings. As these datasets become more ancestrally diverse and better harmonized, they will help mitigate long-standing disparities in variant interpretation that disproportionately affect underrepresented populations.

Collectively, these developments are poised to improve the precision and equity of CS by enabling more confident variant classification, reducing uncertainty for patients and clinicians, and increasing the overall clinical utility and over time stability of screening results worldwide.

## 4. Inclusion of Lethal Genes in ECS Panels: Ethical and Clinical Considerations

An emerging area of interest in the design of expanded carrier screening (ECS) panels is whether to include genes that are lethal in utero or preimplantation-lethal, rather than restricting screening to postnatal disorders. Lethal genes in utero are all those genes whose loss of function, due to monoallelic or biallelic variants, compromises embryonic development at different preimplantation o prenatal stages and are therefore considered incompatible with life. In some cases, we can also refer to pre-implantation lethal genes, in those cases where the arrest occurs even before implantation in utero [16]. Traditionally, ECS has focused on identifying couples at risk for childhood-onset Mendelian diseases. However, current panels largely exclude genes associated with early embryonic lethality, recurrent miscarriage, and infertility endophenotypes, despite increasing evidence of their relevance to reproductive outcomes. Aminbeidokhti et al. (2024) [32] analyzed population-level allele frequencies from gnomAD and identified 138 recessive candidate genes in which pathogenic (P), likely pathogenic (LP), or loss-of-function (LoF) variants occur at frequencies ≥0.5% (≈1/200 individuals), suggesting that potentially lethal alleles are far more common than assumed. These findings intersect with epidemiological observations that nearly 70% of all human conceptions fail, often before clinical detection [33]. Importantly, up to 50% of spontaneous abortions are euploid, indicating that chromosomally normal losses may be driven by monogenic or oligogenic defects rather than aneuploidy.

Further insights come from gene discovery studies. Dawes et al. (2019) proposed that ~600 human genes may contribute to perinatal lethality [34]. Shamseldin et al. (2021) [35] analyzed 481 fetal or early neonatal deaths—largely from consanguineous families—and identified 46 novel lethal genes, including genes previously associated only with dominant conditions but lethal in the homozygous state. However, the evidence base on pregnancy loss genetics remains limited. Most studies are small in size, use heterogeneous methodologies, employ variable sequencing strategies, and often lack systematic phenotyping, making it difficult to draw definitive conclusions or establish robust gene–phenotype associations. This methodological heterogeneity limits reproducibility and reduces the strength of evidence for gene inclusion in population-level screening programs.

The challenge is even greater when considering preimplantation-lethal genes, which contribute to infertility endophenotypes such as oocyte maturation failure, fertilization defects, zygotic genome activation failure, and early embryo arrest. Although several genes implicated in these conditions (e.g., TUBB8, PATL2, WEE2, BTG4, NLRP5) appear to follow recessive inheritance patterns and are biologically plausible candidates for ECS, the supporting literature remains narrow [36]. Most infertility gene studies have been conducted primarily in Asian cohorts, often with unclear criteria for case selection, lack of matched controls, and imprecise phenotyping of oocyte or embryo developmental stages. As a result, their generalizability to diverse populations is limited, and the true prevalence or penetrance of these variants remains uncertain [37].

Given these limitations, the question of whether to incorporate lethal and preimplantation-lethal genes into ECS panels is complex. A more inclusive strategy could:

- Identify more couples at risk for conceptions that fail before clinical pregnancy is established.
- Explain otherwise “idiopathic” infertility or recurrent pregnancy loss.
- Enable targeted reproductive interventions such as preimplantation genetic testing (PGT) to prevent repeated failed cycles or miscarriages.
- Yet, the inclusion of such genes also presents significant challenges:
- Interpretation difficulty, due to limited functional evidence, incomplete penetrance, or uncertain pathogenicity.
- Communication challenges, as couples may struggle to interpret risks related to embryonic outcomes that occur before pregnancy detection.
- Ethical concerns, including potential anxiety, overuse of assisted reproductive technologies, and decisions based on incomplete or population-specific evidence.

Furthermore, the inclusion of genes associated with lethality in utero in expanded carrier screening (ECS) panels raises important challenges for genetic counselling. For many of these conditions, the actual risk and probability of adverse fetal outcomes in carrier couples cannot yet be quantified with precision. Robust penetrance estimates derived from prospective or population-based studies are not available for all genes currently classified as “lethal,” and variant interpretation remains uncertain for a subset of these conditions.

In addition, the clinical impact of a variant often depends on its specific molecular nature (e.g., hypomorphic versus loss-of-function variants), genotype–phenotype correlations, and modifying factors, which may be difficult to convey clearly to couples during counselling. This complexity may limit the usefulness of such information in supporting informed reproductive decision-making [38].

For these reasons, the inclusion of lethal in utero conditions in ECS panels requires careful consideration. Genetic counselling in this context should account not only for the current limits of clinical validity and interpretability, but also for the psychological, ethical, and communicative implications for prospective parents [39]. Future studies are therefore required to strengthen the clinical validity of these findings and to prospectively evaluate the clinical utility and counselling implications of reporting lethal genes in preconception carrier screening. In summary, while expanding ECS to include lethal and preimplantation-lethal genes holds promise for improving reproductive diagnostics, robust population-level data, standardized phenotyping, and high-quality functional studies are essential before such genes can be responsibly incorporated into clinical screening panels.

## 5. The Role of Genetic Counselling in CS Programs

Genetic counselling is a fundamental and mandatory step, as suggested by SIGU recommendations, for carrier screening to truly become a useful tool for couples in their reproductive choices. Counselling should aim to provide a clear understanding of the type of test to be performed, its limitations, the presence of residual risks, the possibility of having to face any clinical consequences, and the hypothetical impact on the entire family. Genetic counselling has the task of informing the couple about any risks without influencing their reproductive choices. Various scientific societies (SIGU, ACMG, and ACOG) agree on the importance and crucial role of genetic counselling, which must be provided by specialized healthcare professionals [8]. The geneticist must also be skilled at adapting communication to different contexts, as the perception of risk and the information received is subjective and influenced by cultural factors. The residual risk must be clearly communicated to the couple, who must understand that the test significantly reduces, but does not eliminate, the possibility of disease in their offspring. In order to calculate the residual risk, both partners must be tested. As suggested by the SIGU recommendations [4], there are two types of approach to ECS testing: simultaneous (both partners undergo testing at the same time) or sequential (testing is extended to the second partner, usually the man, if the first partner tests positive). In pregnancy, the simultaneous approach is useful as it reduces the time required and provides a rapid and comprehensive assessment of reproductive risk. Genetic counselling should also be helpful to the couple in their post-test reproductive choices. In the presence of a positive test result, the couple can evaluate different options, including pre-implantation diagnosis for monogenic diseases (PGT-M) [1], gamete donation and prenatal diagnosis.

Another important challenge is managing counselling in the presence of VUS variants or conflicting variants with a clinical significance that is still unclear. The approach to increasingly extensive panels for CS must therefore be carefully considered and well managed to ensure that this extremely delicate counselling does not create more confusion than benefit for the couple. Face-to-face genetic counselling remains the gold standard, but technological advances have led to the spread of online genetic counselling in recent years. Online counselling can overcome logistical barriers, reduce waiting times, make appointments more flexible and offer couples the opportunity to access services from home. However, to be effective, online counselling must meet certain standards in terms of quality, data protection and communication. It is also essential that it maintains the human and personalized approach that forms the ethical and communicative basis of genetic counselling. A systematic review by Danylchuk (2021) analyzed 42 studies involving a total of 13,901 patients, highlighting that online genetic counselling does not differ in terms of knowledge gained, patient satisfaction or anxiety compared to face-to-face counselling [40]. The use of artificial intelligence is increasingly emerging in all areas of medicine. The use of AI in genetic counselling is taking on a growing role, thanks to the presence of digital counselling support tools. A study by Jeon et al. (2025) [41] evaluated the accuracy of information provided by four generative AI models (ChatGPT 5.1, Gemini, Claude, Perplexity). They were evaluated using 102 questions on rare diseases covering general information, diagnosis, treatment, prognosis and advice, based on the Likert scale. It was concluded that all four AI models provided reliable information. However, they occasionally reported inaccurate information that caused anxiety and confusion in patients. These tools can assist in compiling pedigrees, automatically calculating risk scores, generating standardized reports, preliminarily selecting test candidates, and providing pre-test information. The function of AI is to support, not replace, the genetic counsellor. The introduction of artificial intelligence into clinical practice is constantly evolving. In recent years, machine learning (ML) has demonstrated growing potential as a tool to support genetic diagnosis and counselling. In a recent study [42], the authors developed an ML algorithm as a predictive model based on Random Forest. The model used data extracted from electronic health records (EHRs) to predict the probability that a patient had Fragile X syndrome (FXS) before the actual diagnosis. The use of ML in clinical practice is a useful tool for optimizing diagnosis and interpretation of complex genetic variants, thereby improving the quality of genetic counselling. However, the use of AI and ML in genetic counselling has significant limitations. AI is not always able to associate genomic data with non-genomic data, such as an incomplete family history or the patient’s psychosocial status. Furthermore, ML mechanisms can make it difficult to understand the reasoning behind a result, as they often work like “black boxes”. Although interpretable AI methods are also being developed that will likely streamline the analytical workflows and improve the overall variant annotation accuracy and protocols [43] It is important to emphasize that these tools (IA and ML) must be used in a multidisciplinary context that brings together clinical geneticists and psychologists to ensure that the technology supports doctors and acts as an additional tool for patients and families but does not replace the genetic counsellor.

## 6. Conclusions

Expanded carrier screening has proven clinical value in identifying couples at risk for severe recessive and X-linked conditions, but its optimal implementation requires careful balancing of clinical utility, interpretability, ethical considerations, and counselling needs (Table 2). Importantly, the availability of large, diverse population genomic datasets has enabled a more evidence-based and data-driven approach to ECS panel design, supporting pan-ethnic strategies that are both clinically meaningful and cost-effective compared with ancestry-based screening.

From an economic perspective, multiple cost-effectiveness analyses have consistently demonstrated that carrier screening strategies—whether targeted or expanded—are associated with favorable cost-effectiveness profiles compared with no screening, particularly when contrasted with the long-term medical, social, and economic costs of managing affected children. These studies indicate that upfront investment in screening is generally offset by reductions in healthcare expenditures related to diagnosis, treatment, and lifelong care, supporting the overall economic sustainability of carrier screening programs despite differences in panel size and design [44,45].

At the same time, panel expansion—particularly the inclusion of prenatally lethal genes—raises additional challenges related to penetrance uncertainty, counselling complexity, and psychosocial impact. These factors underscore the central role of high-quality genetic counselling and the need for clear, harmonized guidelines to support responsible reporting and informed reproductive decision-making.

Future efforts should focus on refining gene inclusion criteria, improving prospective data on clinical outcomes, and ensuring equitable access to counselling and screening. Through coordinated action among laboratories, clinicians, researchers, and policymakers, ECS can continue to evolve as a robust, preventive, and ethically grounded tool in reproductive medicine.

## Figures and Tables

**Table 1:** Summary of studies evaluating different gene panels used in expanded carrier screening (ECS).

Authors (Year)	Panel/Number of Genes	Population	Technologies	% At-Risk Couples
Ghiossi et al., J Genet Couns (2018)—Clinical Utility of ECS [10]	110 genes	Not specified (results 537 ARC)	NGS-based panel	2.5%
Guo, M. H., & Gregg, A. R. (2019)—Estimating yields of prenatal carrier screening and implications for design of expanded carrier screening panels [11]	415 genes	Data from gnomAD v2.0.2, based on 123,136 exome sequencing sample	Theoretical model based on Gene Carrier Rate (GCR)	0.17% to 2.52%
Peyser, A. et al. (2019)—Comparing ethnicity-based and expanded carrier screening methods at a single fertility center reveals significant differences in carrier rates and carrier couple rates [12]	104 genes (comparison with ACOG panel/ethnicity)	4232 individuals	NGS-based panel	1.2%
Capalbo et al., (2019)—Optimizing clinical exome design and parallel gene-testing for recessive genetic conditions in preconception carrier screening: Translational research genomic data from 14,125 exomes [13]	114 genes-conditions	14,125 analysis (5845 gamete donors and 8280 infertile patients)	Exome sequencing	4.8%
Tong K. et al. (2022)—“Clinical Utility of Medical Exome Sequencing” (China) [14]	4158 genes	2234 couples	Medical Exome Sequencing	9.80%
Westemeyer, M. et al., (2020)—Clinical experience with carrier screening in a general population: support for a comprehensive pan-ethnic approach [15]	274 genes	381,014 individuals	NGS-based panel	2.28%
Capalbo A. et al., (2021)—Clinical validity and utility of preconception expanded carrier screening for the management of reproductive genetic risk in IVF and general population [16]	20 genes	3877 individuals	NGS based panel and qPCR	2.6%
Strauss, T. S. et al., (2023)—Barriers to completion of expanded carrier screening in an inner city population [17]	283 genes	222 couples	NGS-based panel	9.5%
Mackenzie’s Mission (Kirk, Archibald et al., NEJM—(2024)) [18]	1281 genes (~750 diseases)	National program in Australia—10,038 couples enrolled	Exome sequencing and NGS panel	1.9%
Zhang X, Chen Q, Li J et al.—(2024) The effectiveness of expanded carrier screening based on next-generation sequencing for severe monogenic genetic diseases [19]	147 genes (155 diseases)	1048 couples	NGS-based panel	5.34%
Feldman et al., 2024—Expanded targeted preconception screening panel in Israel [20]	357 genes (1487 variants)	10,115 Israelis (6036 couples tested)	SNP-Array	2.6%
Huang et al., 2024—Comprehensive analysis of NGS-based expanded carrier screening in southern/southwestern China [21]	220 genes	1512 couples	NGS-based panel	Not specified
Tan et al., 2024—Expanded carrier screening for 224 monogenic disease genes in 1499 Chinese couples [22]	224 genes	1499 couples (China)	NGS-based panel	3.67%

**Table 2:** Summary of advantages and challenges of ECS.

Advantages	Challenges
Early identification of reproductive risk	Variable detection rates and residual risk
Expanded reproductive options	Interpretation issues related to variants of uncertain significance (VUS)
Reduction in incidence of severe recessive disorders	Psychological impact and complex reproductive decision-making
More equitable screening when panels are ancestry-independent	Cost and variability in access across healthcare systems
Supports personalized genetic counseling	Ethical and implementation considerations (consent, misinterpretation, integration in routine care)
Reduction in economic burden for recessive diseases healthcare	

## Data Availability

No new data were created or analyzed in this study.
